# 

**Published:** 1884-01

**Authors:** 


					﻿JANUARY, 1884.
T^iRTY-FIRST YEAR.
HULL’S
jotiiab
OF
Tone? a t mw
AAXaxxXa A O.
Guaranteed Circulation .200,000 Copies in 12 Months.
E. H. GIBBS, A.M., M.D., Editor,
No. 21 CLINTON PLACE, EIGHTH 8TEEET, NEW YOKE.
TERMS: $1 a Year.
Single number, 10 cts.
				

